# Supplementary figures and images for: Site selection by geese in a suburban landscape
Source: PeerJ. 2020 Sep 22;8:e9846. doi: 10.7717/peerj.9846 (PMC7518184; doi:10.7717/peerj.9846)

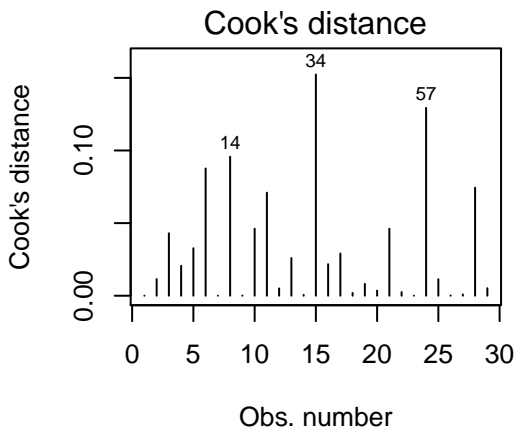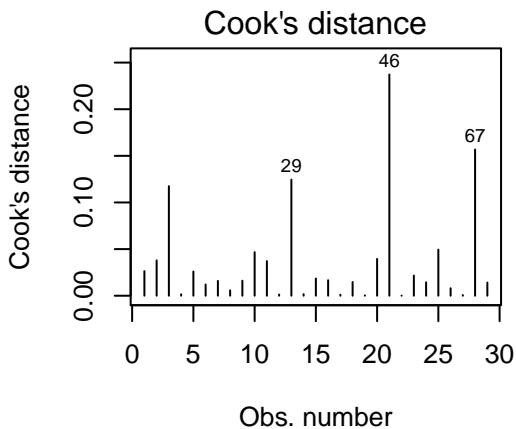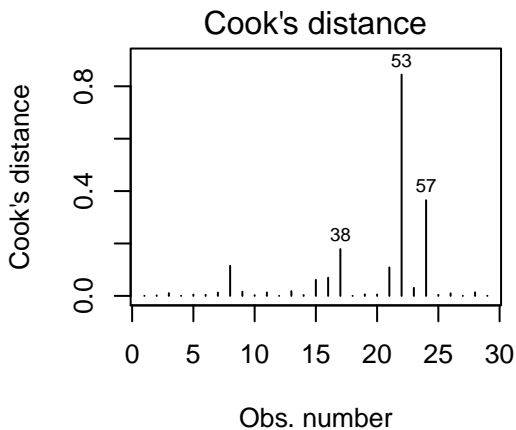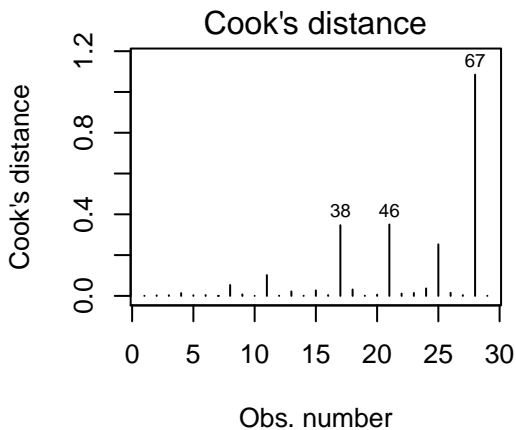

Supplement: Figure S1 — Cook’s distances from minimum adequate models of geese grazing. From right to left and top to bottom. Alopochen aegyptiaca, Anser anser, B. canadensis & B. leucopsis [file peerj-08-9846-s001.pdf]

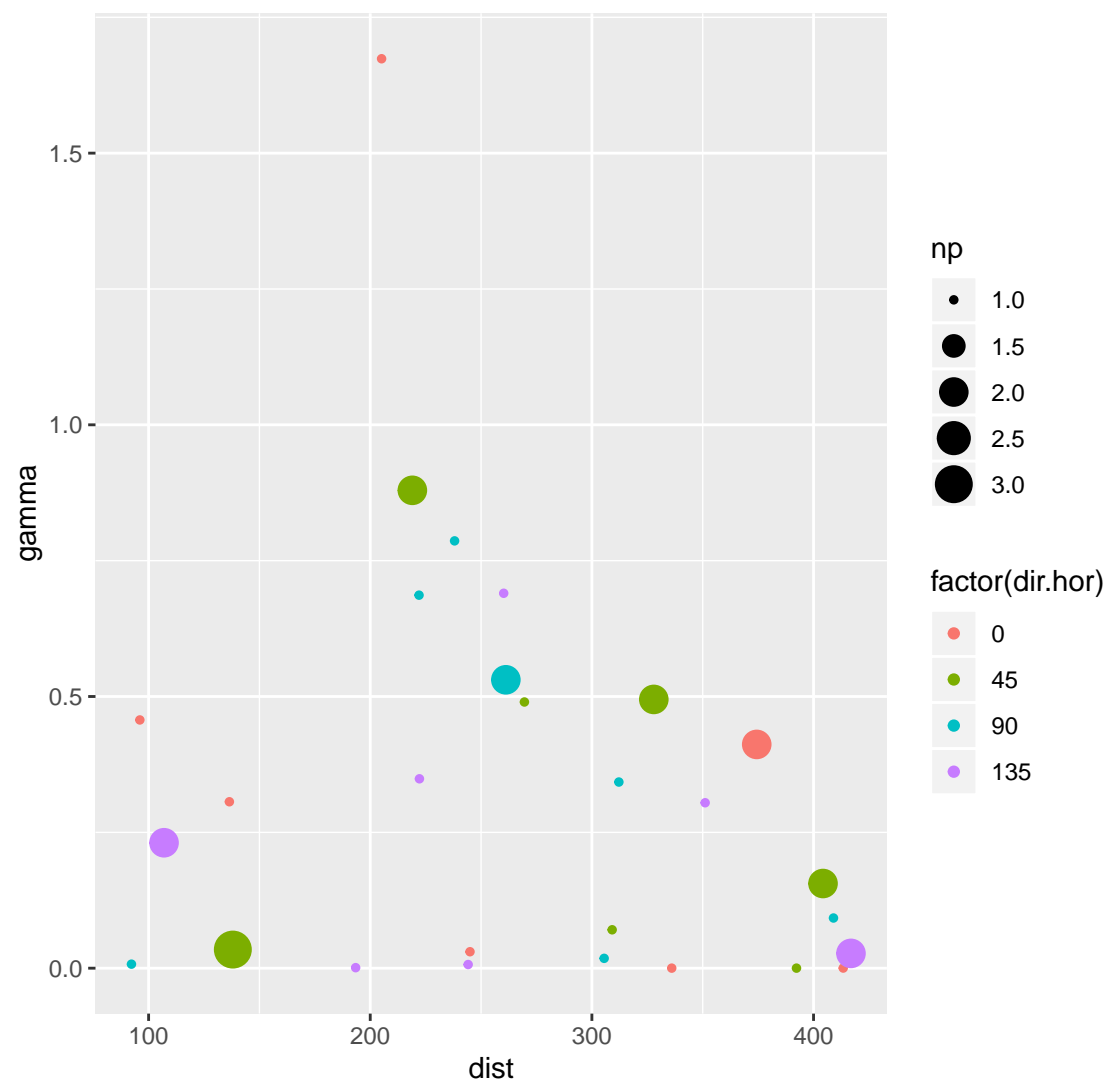

Supplement: Figure S2 — Variogram of model residuals from the minimum adequate model for Alopochen aegyptiaca. The variogram was produced in four directions, np = the number of point pairs for this estimate. [file peerj-08-9846-s002.pdf]

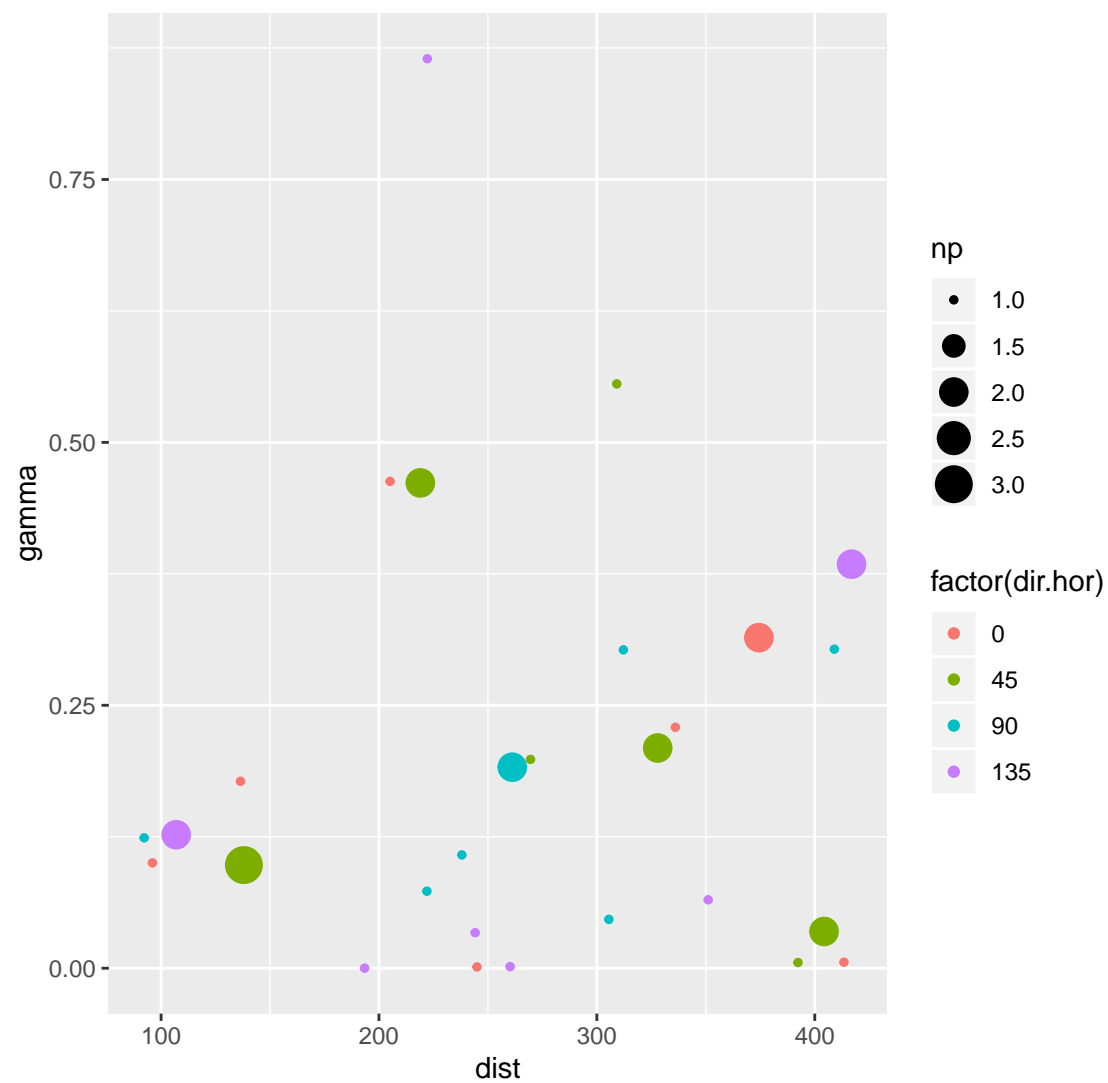

Supplement: Figure S3 — Variogram of model residuals from the minimum adequate model for Anser anser. The variogram was produced in four directions, np = the number of point pairs for this estimate. [file peerj-08-9846-s003.pdf]

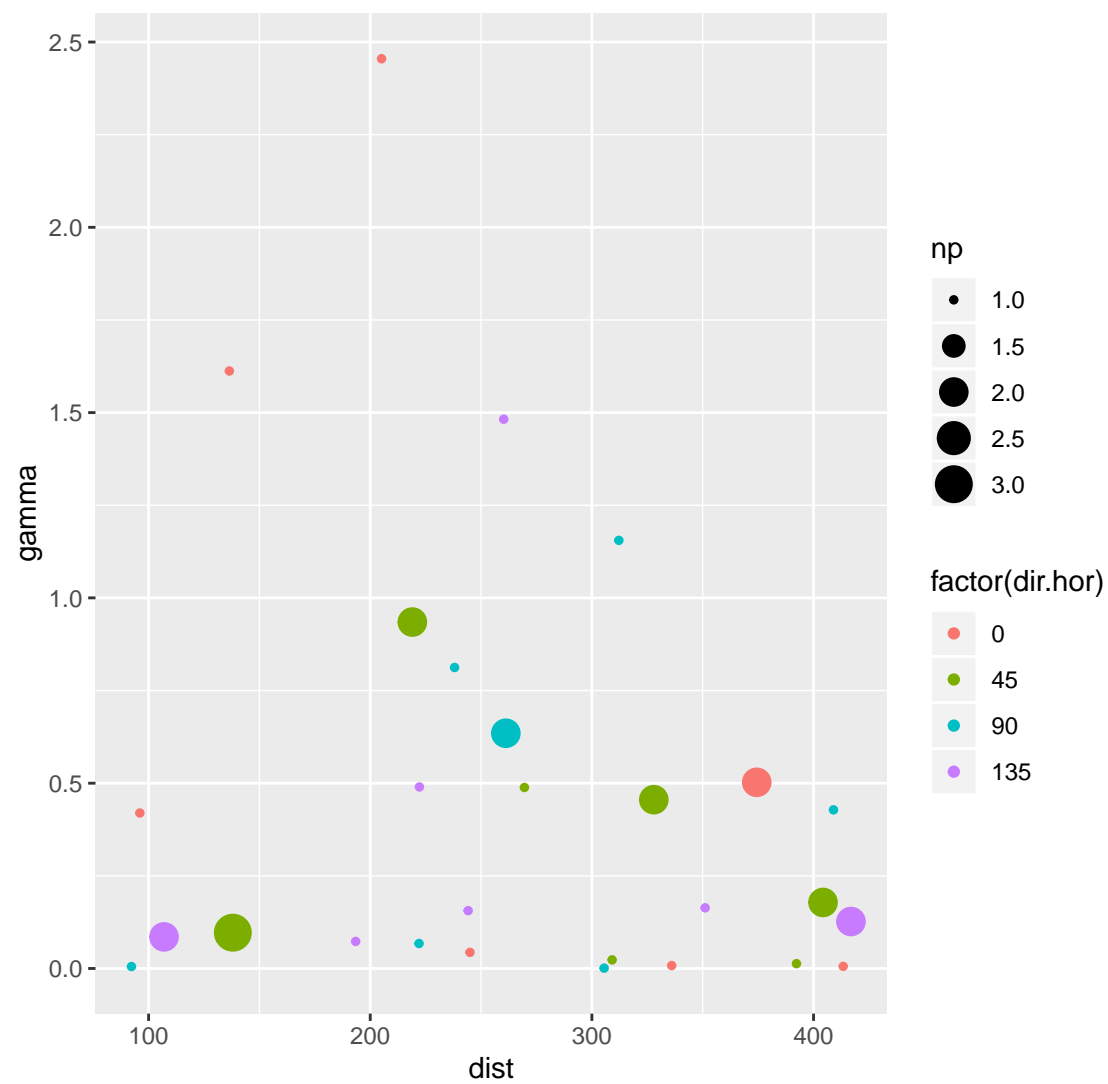

Supplement: Figure S4 — Variogram of model residuals from the minimum adequate model for Branta canadensis. The variogram was produced in four directions, np = the number of point pairs for this estimate. [file peerj-08-9846-s004.pdf]

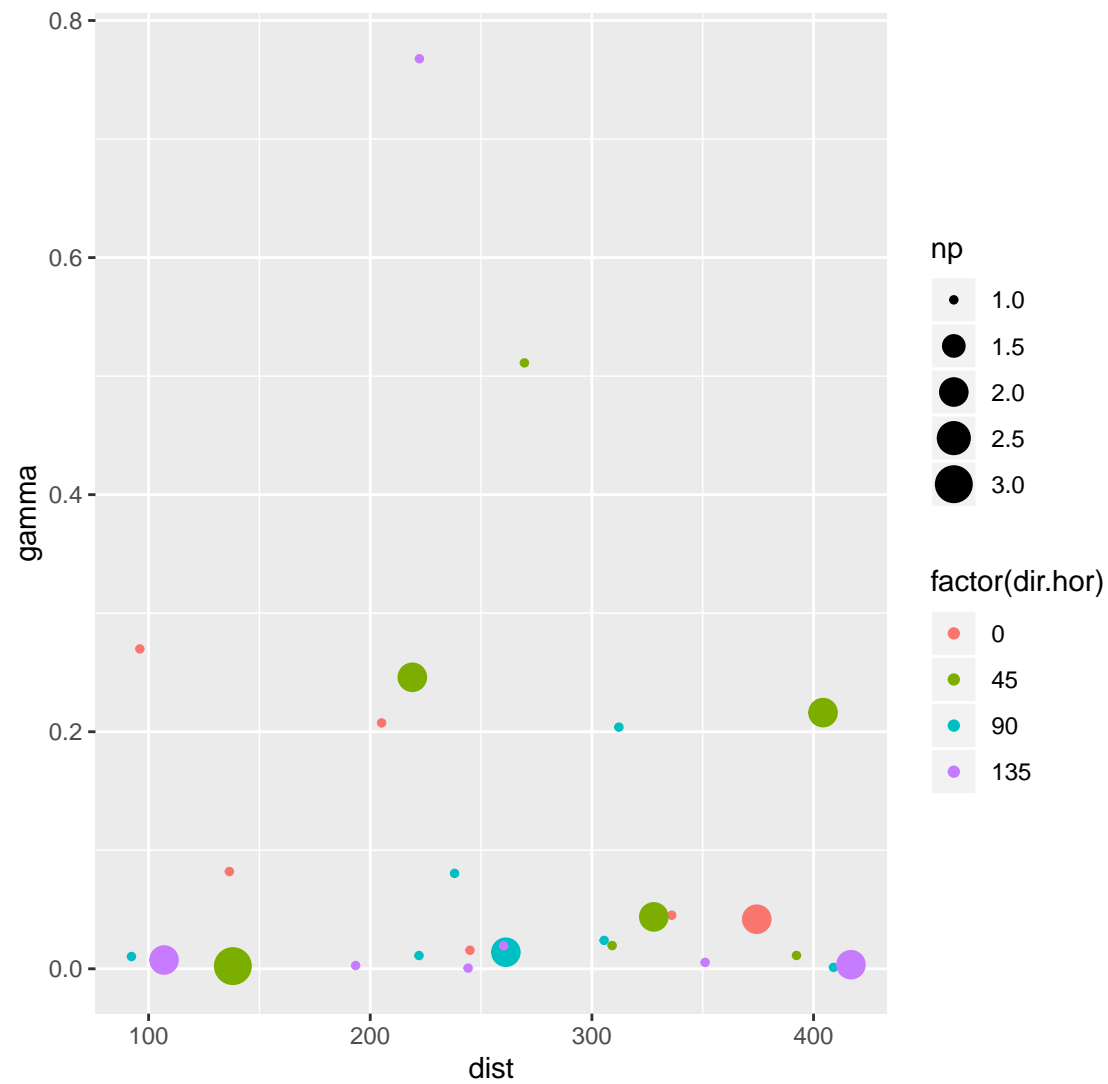

Supplement: Figure S5 — Variogram of model residuals from the minimum adequate model for Branta leucopsis. The variogram was produced in four directions, np = the number of point pairs for this estimate. [file peerj-08-9846-s005.pdf]

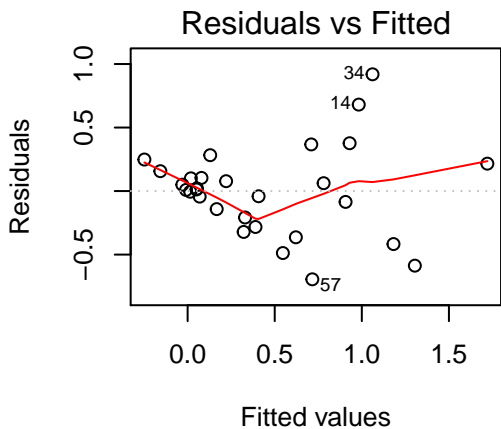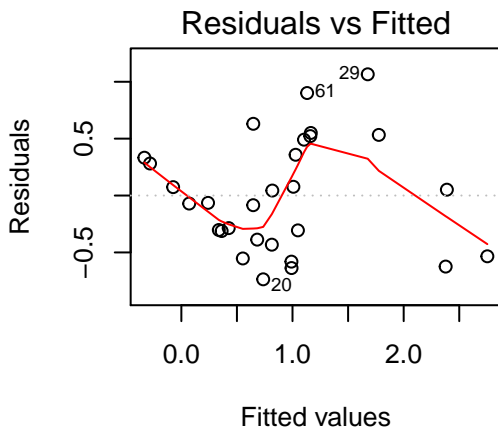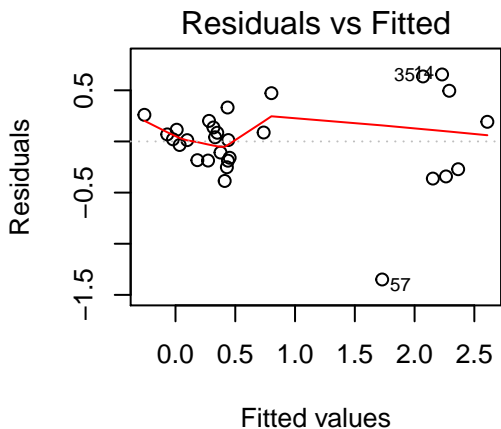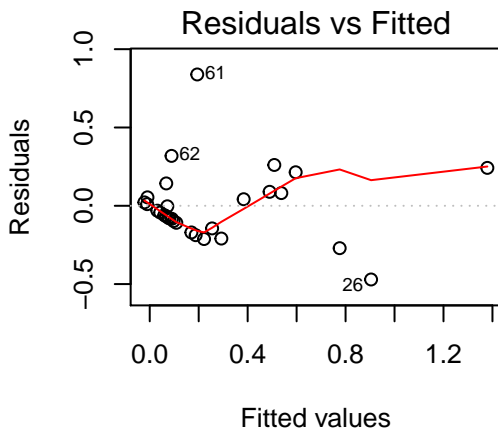

Supplement: Figure S6 — From right to left and top to bottom. Alopochen aegyptiaca, Anser anser, Branta canadensis & Branta leucopsis. [file peerj-08-9846-s006.pdf]

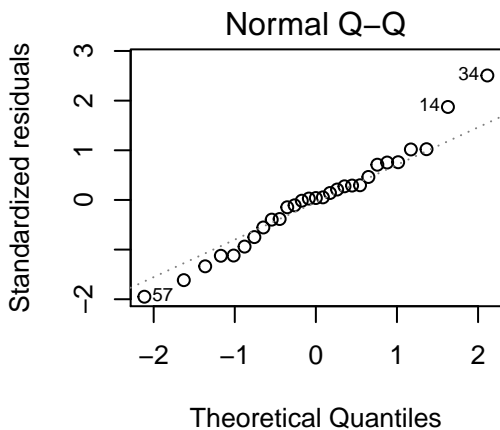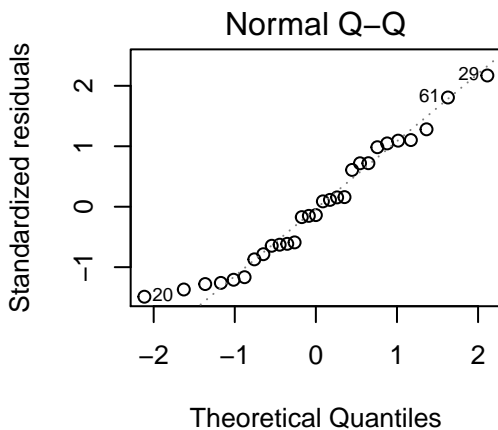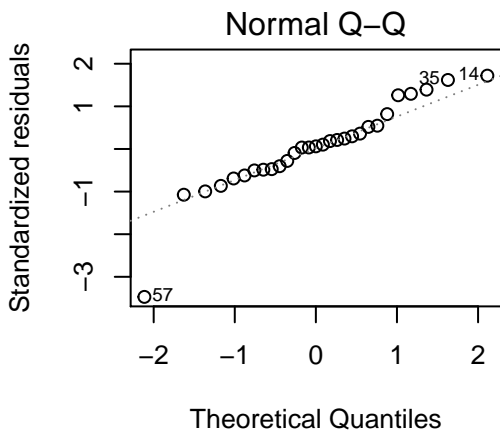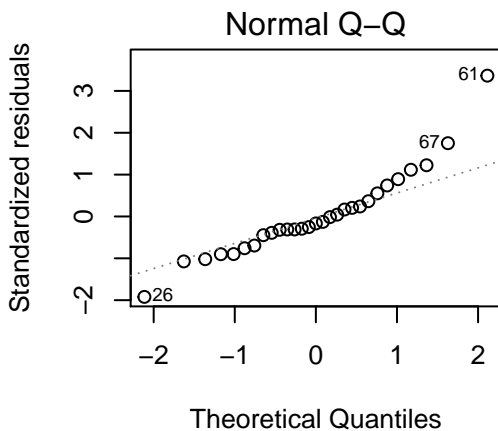

Supplement: Figure S7 — From right to left and top to bottom. Alopochen aegyptiaca, Anser anser, Branta canadensis & Branta leucopsis. [file peerj-08-9846-s007.pdf]

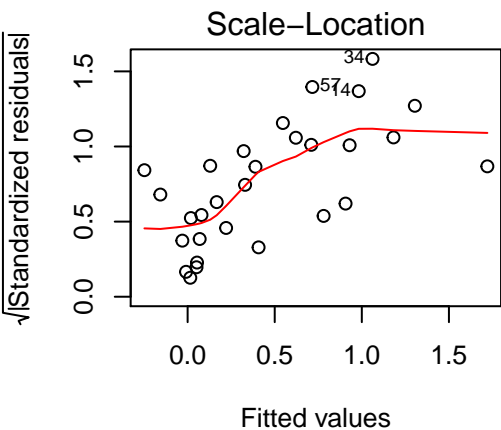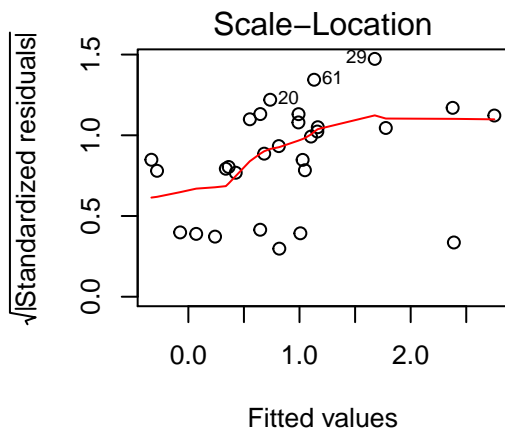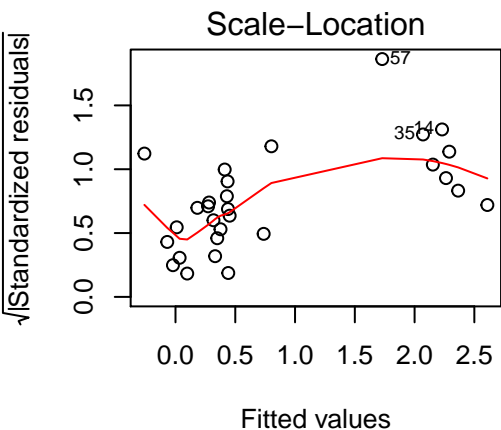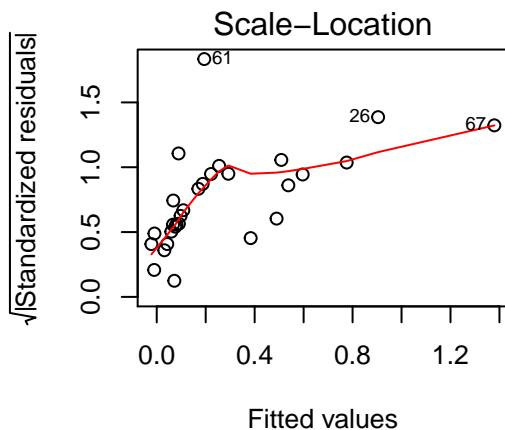

Supplement: Figure S8 — From right to left and top to bottom. Alopochen aegyptiaca, Anser anser, Branta canadensis & Branta leucopsis. [file peerj-08-9846-s008.pdf]
