# Supplementary material for: Site selection by geese in a suburban landscape
Source: PeerJ. 2020 Sep 22;8:e9846. doi: 10.7717/peerj.9846 (PMC7518184; doi:10.7717/peerj.9846)
Supplement: Table S1 — Plots were extended and quadrat numbers increased in the second year to improve the detection of the effects. Plots were chosen with a variety of aspects as noted to avoid any systematic bias related to the direction of the plot with respect to the sun or landscape features. [file peerj-08-9846-s009.docx]

| Plot  number | Sector | Latitude | Longitude | Observer | Date | Dimensions (m) | quadrats | Aspect |
| --- | --- | --- | --- | --- | --- | --- | --- | --- |
| P1 | 67 | 50.927168 | 4.329814 | Colsoulle | 2014-07-07 | 10 x 12 | 20 | S |
| P2 | 67 | 50.926701 | 4.32993 | Colsoulle | 2014-07-09 | 10 x 12 | 20 | N |
| P3 | 67 | 50.92684 | 4.331549 | Colsoulle | 2014-07-10 | 10 x 12 | 20 | S |
| P4 | 67 | 50.926743 | 4.331992 | Colsoulle | 2014-07-11 | 10 x 12 | 20 | S |
| P7 | 14 | 50.92902 | 4.32868 | Delhez | 2015-03-25 | 10 x 15 | 30 | SW |
| P8 | 14 | 50.9294 | 4.32865 | Delhez | 2015-03-30 | 10 x 15 | 30 | SW |
| P9 | 34 | 50.92922 | 4.32663 | Delhez | 2015-03-30 | 10 x 15 | 30 | SE |
| P10 | 34 | 50.92886 | 4.32614 | Delhez | 2015-04-07 | 10 x 15 | 30 | SE |
| P11 | 67 | 50.92644 | 4.33056 | Delhez | 2015-04-08 | 10 x 15 | 30 | SE |
| P12 | 67 | 50.92638 | 4.33093 | Delhez | 2015-04-09 | 10 x 15 | 30 | NW |
