# Supplementary material for: Site selection by geese in a suburban landscape
Source: PeerJ. 2020 Sep 22;8:e9846. doi: 10.7717/peerj.9846 (PMC7518184; doi:10.7717/peerj.9846)
Supplement: Table S2 [file peerj-08-9846-s010.docx]

| lm(formula = log(aegyptiaca + 1) ~ area + distance + woodland +   barriers + area:distance + area:barriers, data = data2019) Residuals:  Min 1Q Median 3Q Max  -0.69472 -0.20809 0.01262 0.15653 0.91915   Residual standard error: 0.3966 on 22 degrees of freedom Multiple R-squared: 0.6628, Adjusted R-squared: 0.5708  F-statistic: 7.207 on 6 and 22 DF, p-value: 0.0002389 | | | |
| --- | --- | --- | --- |
|  | ±S.E. | t | p |
| (Intercept) | 0.578(±0.286) | 2.02 | 0.0556 |
| area | 3.69×10^-5^(±1.46×10^-5^) | 2.51 | **0.020 *** |
| Minimum distance from a lake | 4.12×10^-4^(±1.06×10^-3^) | 0.39 | 0.700 |
| woodland | -0.267(±2.02) | -1.32 | 0.200 |
| barriers to direct flight | -0.613(±0.292) | -2.10 | **0.047 *** |
| area:distance | -2.67×10^-7^(±1.18×10^-7^) | -2.27 | **0.034 *** |
| area:barriers | 5.56×10^-5^(±2.55×10^-5^) | 2.18 | **0.040 *** |
