# Supplementary material for: Site selection by geese in a suburban landscape
Source: PeerJ. 2020 Sep 22;8:e9846. doi: 10.7717/peerj.9846 (PMC7518184; doi:10.7717/peerj.9846)
Supplement: Table S3 [file peerj-08-9846-s011.docx]

| lm(formula = log(anser + 1) ~ area + woodland, data = data2019) Residuals:  Min 1Q Median 3Q Max  -0.73629 -0.38745 -0.06434 0.35822 1.06562  Residual standard error: 0.51 on 26 degrees of freedom Multiple R-squared: 0.6983, Adjusted R-squared: 0.6751  F-statistic: 30.09 on 2 and 26 DF, p-value: 1.714e-07 | | | |
| --- | --- | --- | --- |
|  | ±S.E. | t | p |
| (Intercept) | 0.307(±0.171) | 1.79 | 0.085 |
| area | 5.99×10^-5^(±9.73×10^-6^) | 6.16 | **1.65×10^-6^***** |
| woodland | -0.786(±0.240) | -3.28 | **0.003**** |
