# Supplementary material for: Site selection by geese in a suburban landscape
Source: PeerJ. 2020 Sep 22;8:e9846. doi: 10.7717/peerj.9846 (PMC7518184; doi:10.7717/peerj.9846)
Supplement: Table S4 [file peerj-08-9846-s012.docx]

| lm(formula = log(canadensis + 1) ~ area + distance + woodland +   barriers + distance:barriers, data = data2019)  Residuals:  Min 1Q Median 3Q Max  -1.34943 -0.18677 0.02133 0.19197 0.65493  Residual standard error: 0.4227 on 23 degrees of freedom Multiple R-squared: 0.8498, Adjusted R-squared: 0.8171  F-statistic: 26.02 on 5 and 23 DF, p-value: 9.323e-09 | | | |
| --- | --- | --- | --- |
|  | ±S.E. | t | p |
| (Intercept) | 2.13(±0.273) | 7.82 | **6.40×10^-8^***** |
| area | 1.63×10^-5^(±9.46×10^-6^) | 1.72 | **0.099** |
| distance | -5.75×10^-3^(±1.09×10^-3^) | -5.29 | **2.29×10^-5^***** |
| woodland | -0.338(±0.211) | -1.60 | **0.123** |
| barriers | -1.87(±0.312) | -5.98 | **4.23×10^-6^***** |
| distance:barriers | 5.64×10^-3^(±1.41×10^-3^) | 4.00 | **5.68×10^-4^***** |
