# Supplementary material for: Site selection by geese in a suburban landscape
Source: PeerJ. 2020 Sep 22;8:e9846. doi: 10.7717/peerj.9846 (PMC7518184; doi:10.7717/peerj.9846)
Supplement: Table S5 [file peerj-08-9846-s013.docx]

| lm(formula = log(leucopsis + 1) ~ area + distance + barriers +   area:distance + area:barriers + distance:barriers + area:distance:barriers,   data = data2019)  Residuals:  Min 1Q Median 3Q Max  -0.47063 -0.10868 -0.04301 0.08108 0.83859  Residual standard error: 0.2706 on 21 degrees of freedom Multiple R-squared: 0.6551, Adjusted R-squared: 0.5402  F-statistic: 5.699 on 7 and 21 DF, p-value: 0.0008578 | | | |
| --- | --- | --- | --- |
|  | ±S.E. | t | p |
| (Intercept) | 6.24×10^-3^(±0.287) | 0.02 | 0.983 |
| area | 4.37×10^-5^(±1.46×10^-5^) | 2.99 | 0.007** |
| distance | 1.81×10^-3^(±1.49×10^-3^) | 1.21 | 0.239 |
| barriers | 7.52×10^-2^(±0.342) | 0.22 | 0.828 |
| area:distance | -3.18×10^-7^(±1.29×10^-7^) | -2.47 | 0.022* |
| area:barriers | -5.98×10^-5^(±3.38×10^-5^) | -1.77 | 0.091 |
| distance:barriers | -1.95×10^-3^(±1.70×10^-3^) | -1.15 | 0.264 |
| area:distance:barriers | 4.01×10^-7^(±1.74×10^-7^) | 2.30 | 0.032* |
